# Slovak Local Ewe’s Milk Lump Cheese, a Source of Beneficial *Enterococcus durans* Strain

**DOI:** 10.3390/foods10123091

**Published:** 2021-12-13

**Authors:** Andrea Lauková, Martin Tomáška, Vladimír Kmeť, Viola Strompfová, Monika Pogány Simonová, Emília Dvorožňáková

**Affiliations:** 1 Institute of Animal Physiology, Centre of Biosciences of the Slovak Academy of Sciences, Šoltésovej 4-6, 040 01 Košice, Slovakia; kmetv@saske.sk (V.K.); strompfv@saske.sk (V.S.); simonova@saske.sk (M.P.S.); 2 Dairy Research Institute, a.s., Dlhá 95, 010 01 Žilina, Slovakia; tomaska@vumza.sk; 3 Parasitological Institute of the Slovak Academy of Sciences, Hlinkova 3, 040 01 Košice, Slovakia; dvoroz@saske.sk

**Keywords:** bioactivity, *Enterococcus durans*, safety, ewe’s milk lump cheese

## Abstract

Slovak ewe’s milk lump cheese is produced from unpasteurized ewe’s milk without any added culture. Because of the traditional processing and shaping by hand into a lump, this cheese was given the traditional specialty guaranteed (TSG) label. Up till now, there have existed only limited detailed studies of individual microbiota and their benefits in ewe’s milk lump cheese. Therefore, this study has been focused on the beneficial properties and safety of *Enterococcus durans* strains with the aim to contribute to basic dairy microbiology but also for further application potential and strategy. The total enterococcal count in cheeses reached 3.93 CFU/g (log 10) ± 1.98 on average. Based on a MALDI-TOF mass spectrometry evaluation, the strains were allotted to the species *E. durans* (score, 1.781–2.245). The strains were gelatinase and hemolysis-negative (γ-hemolysis) and were mostly susceptible to commercial antibiotics. Among the strains, *E. durans* ED26E/7 produced the highest value of lactase enzyme β-galactosidase (10 nmoL). ED26E/7 was absent of virulence factor genes such as *Hyl* (hyaluronidase), *IS 16* element and gelatinase *(GelE)*. To test safety, ED26E/7 did not cause mortality in Balb/c mice. Its partially purified bacteriocin substance showed the highest inhibition activity/bioactivity against Gram-positive indicator bacteria: the principal indicator *Enterococcus avium* EA5 (102,400 AU/mL), *Staphylococcus aureus* SA5 and listeriae (25,600 AU/mL). Moreover, 16 staphylococci (out of 22) were inhibited (100 AU/mL), and the growth of 36 (out of 51) enterococcal indicators was as well. After further technological tests, *E. durans* ED26E/7, with its bacteriocin substance, can be supposed as a promising additive to dairy products.

## 1. Introduction

Ewe’s milk and cheeses made from it have a high nutritive value, which has led to high demand for those products that is increasing worldwide [1,2]. The indigenous microbiota in raw milk plays an important role in the cheese formation quality [3]. In general, cheese is composed of microbiota originating from the raw ingredients used, the environment and in some types of cheese, the added starter cultures or adjunct cultures. These many sources of microbiota cause considerable variability in the microbiome across cheese varieties [4]. Based on newly developed modern identification techniques, the microbiota of cheeses can be studied in more detail [4,5,6]. Recently, Parente et al. [7] reported a review study associated with the microbiota of dairy milk. Based on meta-analysis results, it was concluded that four phyla, such as Firmicutes, Proteobacteria, Bacteroidetes and Actinobacteria, included the majority of the most abundant and prevalent taxa identified in milk. There were detected psychrotrophs, bacteria associated with teat skin, with gut and also potentially beneficial lactic acid bacteria (LAB) [4,5,6,7]. In general, LAB are considered food-grade bacteria that are safe to consume. Among LAB, the representatives of the genus *Enterococcus* are found in many food products; these foods are animal-derived, e.g., dairy products [8,9,10]. The most frequently detected enterococcal species in cow, goat and ewe raw milk are *Enterococcus faecium*, *E. faecalis*, *E. hirae* and/or *E. durans* [7,8,9,10]. The safety approach for enterococci is controversial; they are considered as indicators of a hygiene condition [11,12]; but on the other side, many enterococcal strains possess beneficial properties, and they are even able to produce antimicrobial substances, enterocins, which also showed several beneficial effects in food [13,14,15] and/or influences in food-producing animals [16,17]. Lauková et al. [16] reported the antimicrobial activity of Ent M in feces and caecum of broiler rabbits against pseudomonads and coliforms (*p* < 0.05), and also higher average daily weight gains were detected. Using Ent 2019 and its producing strain *E. faecium* EF2019 = CCM7420, a tendency to modulate the serum biochemistry parameters and to improve immunity, jejunal morphometry, weight gains, feed conversion and meat quality was reported [17]. The most frequently studied from this aspect are strains of the species *E. faecium* [16,17,18]. However, representatives of *E. durans* species have begun to be studied as beneficial microorganisms [19,20]. *E. durans* is grouped in the *E. faecium* group/cluster based on gene similarity 16S rRNA analysis [21]. Similarly, formerly mentioned, the most frequently present enterococcal species in dairy products are involved in this cluster. Some *E. durans* strains are able to produce bacteriocins—durancins [20,22,23,24]. Yanagida et al. [20] described durancin L28-1A as a new bacteriocin from *E. durans* L28-1 isolated from soil. It shows antibacterial activity against lactobacilli, lactococci, carnobacteria and *Weissella* spp. Durancin TW-49M was characterized as a novel non-pediocin-like Class II bacteriocin produced by *E. durans* QU49 from carrot [22]. El Quardy Khay et al. [23] described bacteriocin-producing strain *E. durans* E204 from camel milk. Anti-listerial activity in ham due to bacteriocin produced by *E. durans* 152 from a floor drain sample was reported by Lihui Du et al. [24].

Slovak ewe’s milk lump cheese is a popular food article among the population in Slovakia. It is produced from unpasteurized ewe’s milk without any added culture. Ewe’s milk lump cheese should contain at least 47% dry matter and 52% fat in dry matter. Its processing was described in more detail in our previous study [5,25]. The final cheese is slightly acidic. This cheese was given the traditional specialty guaranteed (TSG) label [26] because of the traditional processing and shaping by hand into a lump. However, up till now, the study of individual microbiota in ewe’s milk lump cheese has been looked at in limited detail. Therefore, this study has been focused on *E. durans* strains isolated from ewe’s milk lump cheese and especially on beneficial properties (useful enzymes, bacteriocin activity) and safety of the strain *E. durans* ED26E/7 (e.g., virulence factor gene) with the aim to contribute to basic dairy microbiology but also for its further application as an additive. The novelty of this study lies in the fact that *E. durans* from Slovak local ewe’s milk lump cheese has not been characterized up to now from this aspect. Therefore, knowing its beneficial potential brings a promising perspective for dairy processing, which indicates a practical aspect of the study.

## 2. Materials and Methods

### 2.1. Source of Milk and Cheese Samples

Thirty-eight (38) ewe’s milk lump cheeses were sampled. Cheeses were made from milk of the Valachian breed, the most represented sheep breed in Slovakia [2]. This breed is mostly kept in mountain areas and is considered a multi-purpose breed for milk (cheese) production [27]. Cheese samples were supplied during the main production season by different agrofarms (34) in central Slovakia. The farms raise sheep that are grazed in one flock in a wild pasture all day, and they are kept in enclosures at night and during milking, as previously described by Kováčová et al. [2].

The processing of ewe’s milk to manufacture milk lump cheese has already been described in our previous study [5]. Briefly, milk lump cheese manufacture involves several phases, such as making cheese curd, treating raw material, lump forming and dripping [28]. Each lump is left dripping at a temperature of 18–20 °C for 10–24 h and placed on a shelf at 13–15 °C with sufficient ventilation. After three days, a slight skin covering is formed, and small grain-sized holes are developed inside the cheese. The taste of the final cheese is slightly acidic [28].

### 2.2. Isolation and Identification of Enterococcus durans 

Cheese samples were treated by the standard microbiological method (International Organization for Standardization); 10 g of each cheese sample in 90 mL of Ringer solution (Merck, Darmstadt, Germany) was stirred using the Stomacher–Masticator (Spain) and diluted. The appropriate dilutions were plated on Slanetz-Bartley agar and M-Enterococcus agar (Difco, MD, USA). The plates were incubated at 37 °C for 48 h. Fifty-nine (59) presumed colonies were streaked on Brain heart agar (Difco, MD, USA) enriched with defibrinated sheep blood (5%) to check their purity. Pure colonies were submitted for identification using MALDI-TOF mass spectrometry based on protein “fingerprints“ [29] (MALDI-TOF MS, Bruker Daltonics, Billerica, MA, USA), performed using a Microflex MALDI-TOF mass spectrophotometer previously described by Lauková et al. [30]. Lysates of bacterial cells were prepared according to the producer’s instruction (Bruker Daltonics, Billerica, MA, USA). The results evaluation was performed using the MALDI Biotyper 3.0 (Bruker Daltonics, Billerica, MA, USA) identification database. Taxonomic allocation was evaluated on the basis of highly probable species identification (score 2.300–3.000), secure probable species identification/probable species identification (2.000–2.299) and probable genus identification (1.700–1.999). Positive control was strain involved in the identification system database (*E. durans* DSM 20633T DSM). Identical colonies evaluated by the MALDI-TOF score value were excluded. Identified strains were maintained on M-Enterococcus agar (Difco, MD, USA-ISO 7899) for the next testing and also stored using the Microbank system (Pro-Lab Diagnostic, Richmond, BC, Canada).

### 2.3. Phenotypization and Enzyme Production of E. durans 

Phenotypization of *E. durans* strains was performed based on consensus matrix of tests for identification of *Enterococcus* spp. as reported by Manero and Blanch [31], also involving the Rapid ID Streptotest (R Inc., Reading, PA, USA), as well as the API-ZYM system (BioMérieux, France). The testing panel involves arginine, esculin, mannitol, sorbitol, raffinose, inulin, galactose, glucose, NAG (*N*-acetyl-glucosamine), hippurate hydrolysis, etc. Type strain *E. durans* ATCC 19432 was used as a control. 

The following enzymes were tested using the API-ZYM system: alkalic phosphatase, esterase, esterase lipase (C8), lipase (C14), leucine arylamidase, valine arylamidase, cystine arylamidase, trypsin, α-chymotrypsin, acidic phosphatase, naphtol-AS-B1-phosphohydrolase, α-galactosidase, β-galactosidase, β-glucuronidase, α-glucosidase, β-glucosidase, *N*-acetyl-β-glucosaminidase, α-mannosidase and α-fucosidase. Enzyme activities were evaluated according to the manufacturer’s recommendations (after 4 h of incubation at 37 °C). Color intensity values from 0 to 5 and relevant values in nanomoles (nmoL) were assigned for each reaction according to the color chart supplied with the kit.

### 2.4. Hemolysis, Gelatinase Activities and Antibiotic Profile

Hemolysis was detected by streaking the cultures on MRS agar (Difco) supplemented with 5% of defibrinated sheep blood. Plates were incubated at 37 °C for 24–48 h under semi-anaerobic conditions. The presence or absence of clearing zones around the colonies was interpreted as β-hemolysis and negative γ-hemolysis, respectively [32].

Gelatinase is a proteolytic enzyme (extracellular metalloendopeptidase EC 3.4.24.30) that acts on a variety of substrates, such as insulin-beta chain, collagenous material in tissues, the vasoconstrictor endothelin-1, as well as sex-pheromones and their inhibitor peptide [33]. Gelatinase activity was detected with a 3% gelatin medium (Todd-Hewitt agar, Becton and Dickinson, Cockeysville, MD, USA). After the growth of tested strains (48 h at 37 °C), plates were flooded with a 15% solution (HgCl_2_ in 20% HCl). The loss of turbidity halos around colonies was then checked at 4 °C [34].

The antibiotic phenotype was tested using two methods; the disk diffusion method [35], as well as according to EFSA rules using strips [36] with minimal inhibitory concentration (MIC in µg). In both cases, Mueller–Hinton agar enriched with defibrinated sheep blood was used (Difco). The control strain was *E. faecium* CCM4231 [37]. Antibiotic disks included clindamycin (Da, 2 µg), novobiocin (Nv, 5 µg), ampicillin (Amp, 10 µg), erythromycin, azithromycin (15 µg, E, Azm), streptomycin (S, 25 µg), chloramphenicol, kanamycin, vancomycin, tetracycline and rifampicin (C, Kan, Van, Tct, R, 30 µg). Antibiotic disks were supplied by Becton and Dickinson (Cockeysville, MD, USA) and Oxoid (Lowell, MA, USA) as well. Inhibition zones (in mm) were evaluated according to the manufacturer’s instructions. Following the strip method, the strips used were: ampicillin (0.015–256 µg/mL), penicillin (0.032–34 µg/mL), Tct (0.015–256 µg/mL), E (0.015–256 µg/mL) and Van (0.015–256 µg/mL). The *E. faecalis* strain ATCC 29212 was a positive control.

### 2.5. Structural Enterocin Genes Analysis

To detect structural enterocin genes in *E. durans* strains, the most frequently detected genes for enterocins production were analyzed: Ent A, P, B, L50B [38,39]. The primers for durancin were not available for testing. Primers sequences for PCR amplification of *Ents* genes were used according to Aymerich et al. [40], Casaus et al. [41] and Cintas et al. [42,43]: 5 min denaturation at 95 °C, 30 cycles 30 s at 95 °C, 30 s at 58 °C, 30 s at 72 °C; 5 min at 72 °C, 4 °C. The annealing temperature for *Ent* P, L50B and B was 56 °C instead of 58 °C. The PCR product was visualized by 2% agarose electrophoresis (1 μg ethidium bromide). As positive controls, we used *E. faecium* EK13 = CCM7419 [35] for Ent A, P; *E. faecium* L50 [42,43] for *Ent* L50B and B. Briefly, template (2 µL) was added to 8.75 µL of the reagent mixture, which contained 0.5 µL of each primer, 1 µL of (10 mmoL/L) dNTPs (Invitrogen) and water to a total volume of 50 µL. The sequences of the primer pairs used for PCR amplification of the *Ents* structural genes are as follows: Ent A, F5′-GGT ACC ACT CAT AGT GC AAA-3′, R 5′-CCC TGG AAT TGC TCC ACC TAA-3′; Ent P, F5′-GCT ACG CGT TCA TAT GGT AAT-3′, R5′-TCC TGC AAT ATT CTC TTT AGC-3′;Ent L50B, F5′-ATG GGA GCA ATC GCA AAA TTA-3′, R5′-TAG CCA TTT TTC AAT TTG ATC-3′; Ent B, F5′-CAA AAT GTA AAA GAA TTA AGA TCG-3′, R5′-AGA GTA TAC ATT TGC TAA CCC-3′. DNA (template) was extracted by the rapid alkaline lysis method [44].

### 2.6. Bacteriocin Activity of E. durans

*Ents* genes possessing strains were tested for antimicrobial activity using the quantitative agar diffusion method [45]. For this test, concentrates of the strains were prepared as follows: strains (0.1% pre-inoculum) were inoculated in the Brain heart broth (Difco, MD, USA) overnight at 37 °C. Then they were centrifuged for 30 min at 10,000 × *g* (at laboratory temperature). Supernatants were concentrated 10-fold using Concentrator plus (Eppendorf AG, Hamburg, Germany) to achieve a volume of 4 mL. Their activity was tested against the principal (the most susceptible indicator *Enterococcus avium* EA5, fecal strain of piglet, our laboratory), and against *Listeriae* from different food products (Table 1) *Listeria monocytogenes* CCM4699 (Czech Culture Collection, Brno, Czech Republic), *L. innocua* LMG13568 (University Brussel, Belgium) and *Staphylococcus aureus* SA5 (our strain isolated from mastitis milk). Bacteriocin activity was expressed in arbitrary units per mL, indicating the highest dilution of bacteriocin that can inhibit the growth of the indicator strain. Based on the results of inhibition activity, ED26E/7 was selected to prepare semi-purified bacteriocin (precipitate). Inhibition activity testing was performed in duplicate.

### 2.7. Partial Purification of Bacteriocin ED26E/7 and Activity Testing

At first, partial purification of the bacteriocin substance followed the protocol according to Mareková et al. [39] for Ent P; the activity of 1600 AU/mL against *E. avium* EA5 strain was measured. Therefore, partial purification was performed according to the protocol for durancin 28L [22], there was a modification regarding the temperature and time of cultivation (although the *durancin* gene was not tested). ED26E/7 was cultivated in 500 mL of MRS (Merck, Germany) at 37 °C for 18 h. Then the broth culture was centrifuged (10,000× *g*) for 30 min (min) at 4 °C. The pH of the supernatant was adjusted to 4.2 and filtrated using a 0.45 µm filter (Millipore Corp. Bedford, MA, USA). The supernatant was precipitated with ammonium sulfate (80% saturation) at 4 °C overnight (18 h). After precipitation and centrifuging (10,000× *g*) for 30 min at 4 °C, the precipitate was re-suspended in the minimum volume of phosphate buffer (pH 6.5) and inhibition activity was tested against *E. avium* EA5. Inhibition activity reached 102,400 AU/mL. Moreover, other indicators were used: *S. aureus* SA5, *L. innocua* LMG13568, *L. monocytogenes* (12) from various food products, various staphylococci from ewe’s milk lump cheese and raw goat milk, *E. faecium*, *E. faecalis, E. hirae* from raw goat milk and meat products, *E. thailandicus* from beavers, and fecal strains *E. mundtii* from roe and red deers.

### 2.8. In Vitro Safety Control 

To control safety of *E. durans* ED26E/7, three specific genes for virulence factors were tested. PCR amplification with the primers and conditions used followed the protocols according to Kubašová et al. [46] and Lauková et al. [47]. The genes tested were *GelE* (gelatinase), *hylEfm* (hyaluronidase) and *Is16* (element IS). The PCR products were separated by means of agarose gel electrophoresis (1.2% *w*/*v*, Sigma-Aldrich, Saint Louis, MO, USA) with 1 µL/mL content of ethidium bromide (Sigma-Aldrich) using 0.5× TAE buffer (Merck, Darmstadt, Germany). PCR fragments were visualized with UV light. *E. faecium* P36 (Dr. Semedo-Lemsaddek, University Lisbon, Portugal) was the positive control. The PCRs were carried out in 25 µL volume with a mixture consisting of 1× reaction buffer, 0.2 mmoL/L of deoxynucleoside triphosphate, 3 mmoL MgCl_2_, 1 µmoL/L of each primer, 1 U of Taq DNA polymerase and 1.5 µL of the DNA template, with the cycling conditions as previously reported by Kubašová et al. [42] and Lauková et al. [43].

### 2.9. In Vivo Safety Control

For the in vivo safety control of *E. durans* ED26E/7 strain, pathogen-free eight-week-old male Balb/c mice (VELAZ Prague, Czech Republic) were used. Their weight was around 18–20 g. Mice maintenance conditions were the same as previously reported by Vargová et al. [48]. Mice were kept under a 12-h light/dark regimen at a temperature of 22–24 °C with a humidity of 56%. They were on a commercial diet, and water was available without restriction. Mice were divided randomly into 2 groups: Control (*n* = 10) and Group 1 (*n* = 10). To differ the ED26E/7 strain from other enterococci, its rifampicin-resistant variant was prepared [49]. ED26E/7 was administered *per os* daily at a dose of 10^9^ CFU/mL in a total dose 100 µL. Counts of ED26E/7, as well as other enterococci, were enumerated after standard microbiological dilution of feces and jejunum (jejunum was homogenized using Masticator, Spain) and plated on BHI agar enriched with rifampicin (100 µg), and M-Enterococcus agar. Counts were expressed in CFU/g (log10) ± SD. Sampling occurred at the start of the experiment (*n* = 20) and on days 7 and 30.

## 3. Results

### 3.1. Identification of E. durans Strains

**Figure 1:**
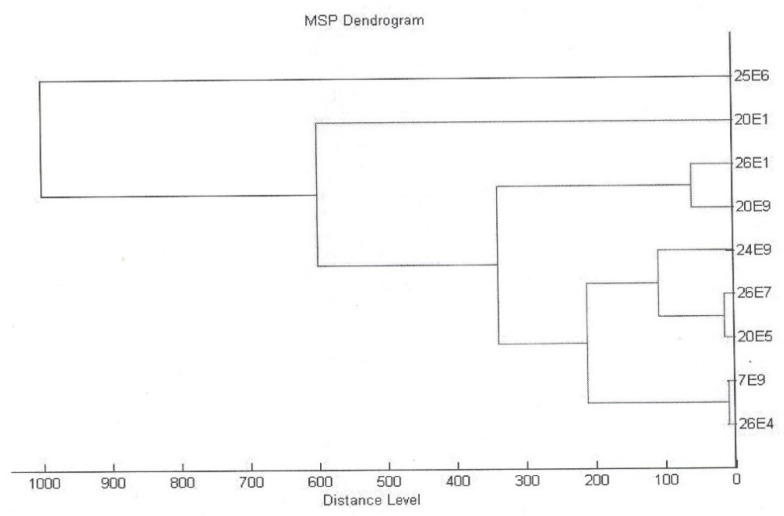
Dendrogram of enterococcal strains from ewe’s milk lump cheese. The vertical axis displays distance between clusters. The horizontal bars indicate the point at which two clusters are merged. (Indicated *E. durans* strains, ED25E/6, ED26E/1, ED7E/9, ED26E/7 and ED24E/9).

The total enterococcal count in the screened cheeses reached 3.93 CFU/g (log 10) ± 1.98 on average. Based on MALDI-TOF mass spectrometry evaluation, strains were allotted to the species *E. durans* with evaluation scores from 1.781 up to 2.245 (Figure 1). Among the nine allotted colonies, five strains (ED24E/9, ED25E/6, ED26E/1, ED26E/7 and ED7E/9) were selected for the next testing. Four identical colonies were excluded. *E. durans* ED26E/7 reached a score of 2.125, ED7E/9 had a score of 1.781, ED26E/1 possessed a score of 1.921, ED25E/6 was scored with a value of 2.245 and ED24E/9 had a score of 2.154. To support taxonomical allotment, phenotyping was provided, and the results were compared with the type strain *E. durans* ATCC 19432. In these five strains, the *N*-acetylglucosamine test was positive, as well as the fermentation of galactose, D-glucose and lactose, while mannitol, inulin and sorbitol were not fermented (they were negative), as well as raffinose. The Voges-Proskauer test was positive; the hydrolysis of esculin showed a dubious reaction and hippurate hydrolysis as well.

*E. durans* strains produced the enzyme alkaline phosphatase at a slight amount, 5 nmoL, similar to lipase, leucine arylamidase, valine arylamidase, cystin arylamidase, α-chymotrypsin, trypsin and acid phosphatase. The enzyme β-glucuronidase was not produced (except for in ED24E/9), and α-mannosidase and α-fucosidase were produced in 5 nmoL or not produced. However, beneficial hydrolase, β-galactosidase was produced by ED26E/7 strain at the amount 10 nmoL; the other strains either produced 5 nmoL or did not produce this enzyme. Esterase, esterase lipase and naphtol-AS-Bi-phosphohydrolase were also produced in amounts of 5 or 10 nmoL. The amount of β-galactosidase was the highest in the ED26E/7 strain (10 nmoL).

### 3.2. Hemolysis, Gelatinase and Antibiotic Phenotype

All *E. durans* strains did not form hemolysis (γ-hemolysis). The gelatinase test was also negative. The strains were susceptible to all antibiotics using the disk diffusion method with inhibition zones ranging from 10 up to 28 mm except for resistance to clindamycin in one strain, novobiocin, streptomycin or kanamycin. However, *E. durans* ED26E/7 was susceptible to all antibiotics with an inhibition size ranging from 13 up to 28 mm. The antibiotic profile of this strain was also evaluated using an E-test. *E. durans* ED26E/7 was susceptible to penicillin (8 µg/mL), ampicillin (2 µg/mL), tetracycline (5 µg/mL), erythromycin and vancomycin (4 µg/mL). MIC breakpoints (µg/mL) for these antibiotics are: P, S ≤ 8/R ≥ 16; Amp, S ≤ 4/R ≥ 16; TCT, S ≤ 4/R ≥ 16;E, S ≤ 0.5/R ≥ 8; Van, S ≤ 4/R ≥ 32.

### 3.3. Structural Genes, Bacteriocin Activity

*Ent A* and *Ent P* genes were detected in tested *E. durans* strains. *Ent P* genes were found in the strains *E. durans* ED25E/6, ED26E/1, ED7E/9 and ED26E/7, and the *Ent A* gene was detected in the strain ED24E/9. ED24E/9 and ED26E/1 did not show inhibition activity against the indicators used. Inhibition activity of strain concentrates ED25E/6, ED7E/9 and ED26E/7 are shown in Table 1. The principal indicator *E. avium* was inhibited by concentrates of the strains ED26E/7 and ED7E/9 with inhibition activities of 800 ± 28.2 and 100 ± 12.1 AU/mL. *L. monocytogenes* CCM4699 was inhibited by concentrates of all three ED strains (100 AU/mL). The other listeriae (five strains) were not inhibited by ED25E/6, and ED7E/9 concentrates; however, concentrate ED26E/7 inhibited the growth of three listeriae (100 ± 10.0, 100 ± 12.2, 100 ± 12.0 AU/mL).

### 3.4. Activity of Partially Purified Bacteriocin—ED26E/7

The highest inhibition activity of the bacteriocin substance ED26E/7 was reached using a purification protocol for durancin L28 [20]. The activity 102,400 ± 320.0 AU/mL was measured; in the first protocol (Materials and Methods), it was only 1600 ± 40.0 AU/mL against the EA5 strain. Ninety-four different indicators were used to test the activity of PPB (partially purified bacteriocin); 73 Gram-positive enterococci and staphylococci, and 21 Gram-negative *E. coli*. Fifty-one enterococcal species strains (*E. faecium, E. faecalis, E. hirae, E. mundtii* and *E. thailandicus*) were used and 22 staphylococcal species strains (*S. aureus, S. simulans, S. xylosus, S. capitis, S. warneri, S. sciuri, S. arlettae, S. schleiferi, S. hominis* and *S. delphini*) from ewe’s milk lump cheeses and from raw goat milk. *S. aureus* SA5 (from mastitis) was inhibited with activity 25,600 ± 116.6 AU/mL (Table 2a); *Listeria innocua* LMG13568 (25,600 ± 160.0 AU/mL), 12 strains of *L. monocytogenes* from different products (25,600 AU/mL, see Table 2a), then 5 strains of *E. thailandicus* from beavers with activity 12,800 AU/mL (Table 2b), fecal *E. mundtii* strains from roe and red deers (7) were inhibited with inhibition activity in the range from 100 up to 400 AU/mL (Table 2b), 9 strains of *E. faecium* from fermented meat products (400–6400 AU/mL, Table 2b,c), 9 *E. faecium* from raw goat milk (200–102,400 AU/mL, Table 2c), 2 strains of *E. faecalis* from meat products (3200, 6400 AU/mL) and 4 strains of *E. hirae* (3 from the meat products and 1 from raw milk) with activity 200–6400 AU/mL. Using 22 staphylococcal strains isolated from raw goat milk, only four strains were inhibited (100 AU/mL); 12 strains were from cheeses (Table 2d). Twenty-one fecal *Escherichia coli* from piglets were tested; however, they were not inhibited.

### 3.5. In Vitro and In Vivo Safety Control (Virulence Factor)

ED26E/7 did not possess genes for *hyl* and *IS 16* elements. It also did not possess the *GelE* gene. ED26E/7 survived well in feces of Balb/c mice; at day 30, it reached almost 10^5^ CFU/g (log 10) (4.61 ± 0.14 CFU/g). It does not cause the mortality of mice.

## 4. Discussion

Enterococcal counts in ewe’s milk cheese were similar as, e.g., in fermented meat products [50], but a lower count (1.82 CFU/mL, log10) was detected in raw goat milk [5]. Lihui Du et al. [24] isolated anti-listerial bacteriocin, the durancin GL producer of which *E. durans* 41D was isolated from Hispanic style cheese. In this study, three out of five identified strains showed identification scores corresponding to secure genus identification/probable species identification (ED26E/7, ED25E/6, ED 24E/9); the scores of the strains ED7E/9 and ED26E/1 corresponded with probable genus identification. However, phenotypic biochemical testing supported characteristics for the species *E. durans* [31].

To evaluate strains as safe, no antibiotic resistance and/or virulence factor genes can be present, or their phenotype has to be assessed, as well as no or slight production of damaging enzymes (disease markers). The production of intestinal disorder enzymes, such as β-glucuronidase, α-chymotrypsin or β-glucosidase, was slight in the *E. durans* tested. *N*-acetyl-β-glucosaminidase was negative or only 5 nmoL; that one enzyme is required to proliferate in vivo [51]. The tested strains seem to be safe regarding enzyme evaluation. In addition, ED26E/7 produced beneficial lactase (β-galactosidase) at the value of 10 nmoL; this enzyme is used in the dairy industry for the production of lactose-free milk to be consumed by lactose-intolerant people [52]. It is known that bacterial β-glucuronidase can play a role in colon cancer; therefore, a zero (0) value for this enzyme measured in *E. durans* strains is also promising from a safety aspect. Enterococci considered for industrial applications should be antibiotic-resistant or have virulence factor genes absent [53]. In general, the production of gelatinase increased the pathogenicity in the animal model [54]. Furthermore, gelatinase can cleave fibrin, which was suggested to have important implications in the virulence of *E. faecalis*, as the secreted protease can damage host tissue and thus allow bacterial migration and spread infection. However, ED26E/7 was hemolysis and gelatinase-negative. The bacteriocin-producing species strain *E. durans* was also isolated from important Egyptian cheese OSY-W [55]. Pieniz et al. [56] described *E. durans* LAB18s isolated from Brazilian soft cheese with the antioxidant activity of its culture supernatant and intracellular extract. Further beneficial/desirable traits may include the production of antioxidants or the expression of pathogens antagonism [55]. Dvorožňáková et al. [57] reported the highest stimulative effect on phagocytosis induced by *E. durans* ED26E/7 strain in mice infected with *Trichinella spiralis*. Moreover, the respiratory burst of blood polymorphonuclear cells was stimulated, which can contribute to a decreased larval migration and destruction of muscle larvae, and therefore, reduced parasite burden in the host. The application of ED26E/7 caused a significant decrease in the number of muscle larvae of *T. spiralis* and showed the highest inhibition effect on female fecundity (94%) [58]. Moreover, the respected increase in macrophage’s metabolic activity induced by ED26E/7 in the intestinal phase of trichinellosis augmented the host-parasite defense (damage and killing newborn larvae with reactive oxygen species from macrophages [48]). The implication of enterococci in food safety was previously reported by Franz et al. [13]. However, the principal condition is that the strain for use has to be safe [59]. In our previous studies, the anti-listerial effect of enterocin CCM4231 was reported in Saint-Paulin cheese or traditional Slovak dairy product “Bryndza” [60,61]. In Saint-Paulin cheese experimentally infected with *L. monocytogenes* Ohio, its initial count of 6.7 (log 10) CFU/g was decreased to 1.9 CFU/g in one week after enterocin addition. In “Bryndza“, bacterial reduction (expressed in order of magnitude) was noted after Enterocin CCM4231 application.

It can be summarized that bioactive *E. durans* ED26E/7 was selected from ewe’s milk lump cheese. This gelatinase and hemolysis-negative (γ-hemolysis) strain, most susceptible to commercial antibiotics producing 10 nmoL lactase enzyme β-galactosidase, represents a promising additive for dairy products. ED26E/7 did not possess virulence factor genes, such as *hyl* (hyaluronidase), the *IS16* element or *GelE* gene. Its partially purified bacteriocin substance inhibited the growth of Gram-positive indicator bacteria; the principal indicator *E. avium* EA5, *S. aureus* SA5 and the other 16 out of 22 staphylococci, listeriae, and also 36 out of 51 enterococcal indicators were inhibited. In the experimental Balb/c mice, ED26E/7 did not cause mortality.

## 5. Conclusions

It can be concluded that *E. durans* ED26E/7 represents a promising beneficial strain for dairy products manufacturing as a natural, bacteriocin-producing adjunct, which can prolong the stability of the products (cheese) by influencing microbiota without negative influence on cheese quality, and with respect for the One Health concept strategy. This study also contributes new alternative approaches for safety assessment in food hygiene/technology and based on previous results, also in feed or food-producing animal production system.

## Figures and Tables

**Table 1:** Concentrated substances of *E. durans* strains and their inhibition activity (AU/mL) ± SD.

Producers			
	ED25E/6	ED26E/7	ED7E/9
EA5	ng	800 ± 28.2	100 ± 12.1
CCM4699	100 ± 10.0	100 ± 12.2	100 ± 12.0
13568	ng	100 ± 12.0	100 ± 10.0
P2024	ng	100 ± 10.0	ng
P2116	ng	100 ± 10.0	100 ± 10.0
P6301	ng	100 ± 10.0	ng
P6501	ng	100 ± 12.0	ng
P3300	ng	100 ± 10.0	100 ± 10.0

*E. durans* ED24E/9 and ED26E/1 were negative; ng—negative; EA5, *Enterococcus avium*; CCM4699, *Listeria monocytogenes,* LMG13568, *L. innocua*, P2024-P3300, *L. monocytogenes*; AU/mL, Arbitrary unit per mL; SD—standard deviation.

**Table 2:** (**a**) Inhibition activity of durancin substance produced by ED26E/7 strain against the principal strains (EA5, SA5) and *Listeria* spp. in arbitrary unit per mL (AU/mL) ± SD. (**b**) Inhibition activity of the durancin substance produced by ED26E/7 against enterococci in arbitrary unit per mL (AU/mL) ± SD. (**c**) Inhibition activity of the durancin substance produced by ED26E/7 against enterococci in arbitrary unit per mL (AU/mL) ± SD. (**d**) Inhibition activity of the durancin substance produced by ED26E/7 against various staphylococci from ewe’s milk lump cheeses and raw goat milk in arbitrary unit per mL (AU/mL) ± SD.

(a)	
Indicator Strains	Activity
EA5	25,600 ± 160.0
SA 5	25,600 ± 116.6
*L. innocua*	25,600 ± 160.0
*L. monocytogenes*	
P2024	25,600 ± 160.0
P7401	25,600 ± 120.0
P7562	25,600 ± 160.0
108111	25,600 ± 160.0
3500	25,600 ± 116.0
5285	25,600 ± 120.0
Ve405	25,600 ± 120.0
7395	25,600 ± 40.0
2116	25,600 ± 120.0
6501	25,600 ± 116.6
7223	25,600 ± 40.0
6301	25,600 ± 120.0
(**b**)	
**Indicator Strains**	**Activity**
ETHc10/1	12,800 ± 35.8
ETHc10/2	12,800 ± 40.0
ETHc12/1	12,800 ± 35.8
ETHc12/2	12,800 ± 40.0
ETTr10/1	12,800 ± 35.8
EM3/166/1	100 ± 10.0
EM4/112/1	200 ± 14.1
EM1/133/1	100 ± 10.0
EM/107/2	400 ± 20.0
EM1/90/2	200 ± 14.0
EM5/114/1	100 ± 10.0
EM6/123/1	100 ± 10.0
EF 1Bs	3200 ± 56.6
EF1Ns	6400 ± 80.0
EF2Sc	3200 ± 56.6
EF2Kal	1600± 40.0
EFKL5	800 ± 28.2
EFPL3	3200 ± 56.6
EFPL4	800 ± 20.0
(**c**)	
**Indicator Strains**	**Activity**
EEPL1	6400 ± 80.0
EEKL2	6400 ± 80.0
EHPL2	6400 ± 69.3
EHTOK1	400 ± 20.0
EHTOK2	1600 ± 40.0
EH21	200 ± 14.2
EF6/2	25,600 ± 160.0
EF10/2	6400 ± 80.0
EF11/1	400 ± 20.0
EF12/1	800 ± 28.2
EF14/2	6400 ± 69.0
EF15/1	3200 ± 56.6
EF16/1	6400 ± 69.0
EF 18/1	51,200 ± 220.0
EF23	102,400 ± 320.0
(**d**)	
**Indicator Strains**	**Activity**
SXOs7/2	12,800 ± 40.0
SXOs2/3	800 ± 28.0
SciOs17/4	100 ± 10.0
Sci Os6/3	12,800 ± 113.0
Sci Os5/1	12,800 ± 40.0
SciOs8/1	200 ± 10.0
SciOs18/1	12,800 ± 35.8
SmiOs17/6	400 ± 20.0
SmiOs14/1	25,600 ± 160.0
SmiOs18/4	100 ± 10.0
SAOs2/1	6400 ± 79.8
SqOs54	100 ± 10.0
Sca31/2	100 ± 12.2
SW39	100 ± 10.0
Sq 40/2	100 ± 10.0
SD30	100 ± 10.0

(a) EA5, *Enterococcus avium*; CCM4699, *Listeria monocytogenes,* LMG13568, *L. innocua*, P2024–6301, *L. monocytogenes*; AU/mL, arbitrary unit per mL, ±SD—standard deviation; (b) ET, *Enterococcus thailandicus* from beavers, EM—*E. mundtii* from roe and red deer. EF—*E. faecium* from various meat products; ±SD—standard deviation; (c) EE, *Enterococcus faecalis* EEPL1-EEKL2) from various meat products, EH, *E. hirae* from raw goat milk and meat products (EHTOK1, EHTOK2, EH21), *E. faecium* from raw goat milk (EF6/2-EF23); ±SD—standard deviation; (d) SX, *Staphylococcus xylosus*, Sci, *S. sciuri*, Smi, *S. simulans*, Sq, *S. equorum*, Sca, *S. capitis*, SW, *S. warneri*, SA, *S. aureus*, SD, *S. delphini*) ±SD—standard deviation.

## Data Availability

The complete data produced in this study are available to research applicants.
